# Persistent effects of the Yellow River on the Chinese marginal seas began at least ~880 ka ago

**DOI:** 10.1038/s41598-017-03140-x

**Published:** 2017-06-06

**Authors:** Zhengquan Yao, Xuefa Shi, Shuqing Qiao, Qingsong Liu, Selvaraj Kandasamy, Jianxing Liu, Yanguang Liu, Jihua Liu, Xisheng Fang, Jingjing Gao, Yanguang Dou

**Affiliations:** 1 Key Laboratory of Marine Sedimentology and Environmental Geology, First Institute of Oceanography, State Oceanic Administration, Qingdao, 266061 China; 2 Laboratory for Marine Geology, Qingdao National Laboratory for Marine Science and Technology, Qingdao, 266061 China; 3 Southern University of Science and Technology, Shenzhen, 518055 China; 4 State Key Laboratory of Marine Environmental Science and Department of Geological Oceanography, Xiamen University, Xiamen, 361005 China; 5 Qingdao Institute of Marine Geology, Qingdao, 266071 China

## Abstract

The Yellow River (or Huanghe and also known as China’s Sorrow in ancient times), with the highest sediment load in the world, provides a key link between continental erosion and sediment accumulation in the western Pacific Ocean. However, the exact age of its influence on the marginal sea is highly controversial and uncertain. Here we present high-resolution records of clay minerals and lanthanum to samarium (La/Sm) ratio spanning the past ~1 million years (Myr) from the Bohai and Yellow Seas, the potential sedimentary sinks of the Yellow River. Our results show a climate-driven provenance shift from small, proximal mountain rivers-dominance to the Yellow River-dominance at ~880 ka, a time period consistent with the Mid-Pleistocene orbital shift from 41-kyr to 100-kyr cyclicity. We compare the age of this provenance shift with the available age data for Yellow River headwater integration into the marginal seas and suggest that the persistent influence of the Yellow River on the Chinese marginal seas must have occurred at least ~880 ka ago. To our knowledge, this study provides the first offshore evidence on the drainage history of the Yellow River within an accurate chronology framework.

## Introduction

Earth’s geomorphological features, especially surface drainage patterns, are sculpted by large river systems^1^, such as the Amazon, Ganges-Brahmaputra, Yangtze and Yellow Rivers over tectonic-millennial timescales. The interactions of such large river systems with marginal seas largely regulate biogeochemical cycles^2, 3^, primary productivity^4^ and marine sedimentary formation^5^ in the continental shelves. The Yellow River originates from the Tibetan Plateau in northwestern China, charring extremely high silty sediments. This world’s top turbid river discharges over 1.0 billion tons of sediments to the marginal seas annually^6^ and therefore, it offers a critical linkage between the continental erosion and subsequent sediment accumulation in the western Pacific marginal sea. Large river-marginal sea interaction is thus important for understanding the linkages between the tectonic-induced denudation and Earth’s climate^7, 8^. However, the drainage history of the Yellow River, especially when it began to influence the Chinese marginal seas, is highly controversial, with estimates ranging from the Eocene to the late Pleistocene^9–12^. Results from the integrated geological study suggested that the Yellow River drained eastward directly to the ocean in the Eocene^12^. Differently, Craddock *et al*.^9^ suggested that the appearance of the Yellow River and headward basin integration had occurred during the period between ~1.8 and ~0.5 Myr^9^. Jiang *et al*.^10^ and Kong *et al*.^11^ reported the final integration of the Yellow River with the marginal sea at ~150 ka and ~1.3–1.4 Ma, respectively^10, 11^. Mechanisms underlying the final integration of the Yellow River have been broadly criticized^9, 11, 12^, including climate-driven expansion of lake systems^9^, flooding events^11^ and/or adjustment of river drainage due to tectonic-forcing^12^. Most available evidence on the drainage history of the Yellow River come from geomorphological features, such as fluvial terrace, with the assumption that it must have formed by the Yellow River^9, 11, 13^. Nevertheless, the fluvial incision in the upper and middle reaches might be an adjustment of the local drainage system, and cannot be necessarily related to the final integration of the Yellow River with the marginal seas.

Sediments in the Chinese marginal seas delivered by the Yellow River may contain authentic mineralogical and geochemical evidence and thus could provide direct insights into the final integration and subsequent evolution of the Yellow River, especially the Bohai Sea and Yellow Sea which serves as the potential sedimentary sinks of the Yellow River. Although the modern Yellow River flows into the Bohai Sea, the Yellow River also shifted its lower course several times across the southern Yellow Sea during the late Quaternary^14^. Yang *et al*.^15^ attributed a provenance change at the depth of 233 m in a core retrieved from the Yellow River delta to the final integration of the Yellow River^15^, but without reliable age data to constrain this change.

**Figure 1:**
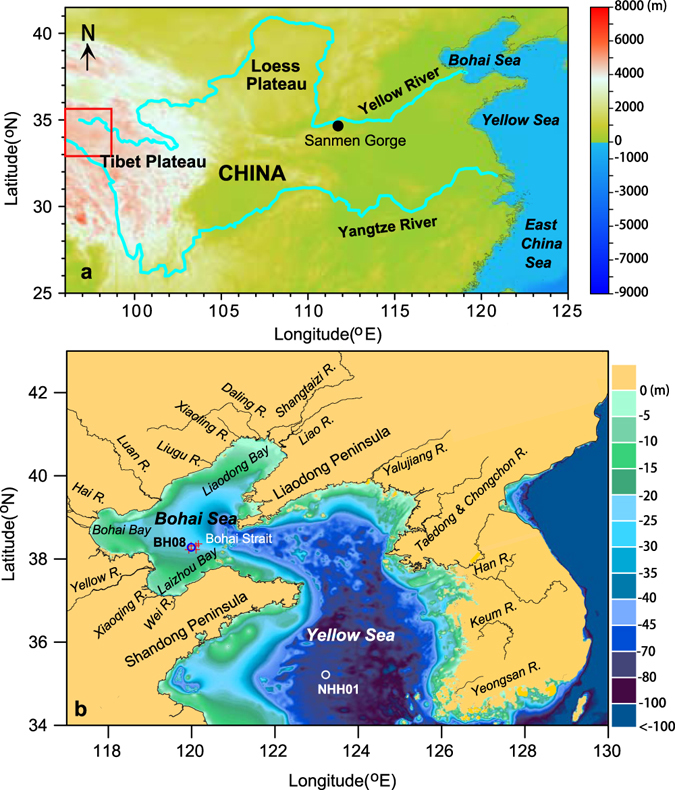
Map showing the Yellow River and location of sediment cores (circles) investigated in this study. (**a**) Geomorphology of China showing the entire course of the Yellow River. (**b**) Locations of core BH08 in the Bohai Sea and core NHH01 in the Yellow Sea, as well as main rivers flowing into the Bohai Sea. Surface samples^30^ close to the core BH08 site (red crosses) were selected to examine the clay minerals for comparison. The red square in panel (**a**) indicates the origin region of the Yellow River and Yangtze River. The base map (**a**,**b**) was generated using the software Surfer (V.13; http://www.goldensoftware.com) and DEM data from http://www.ngdc.noaa.gov/mgg/.

**Figure 2:**
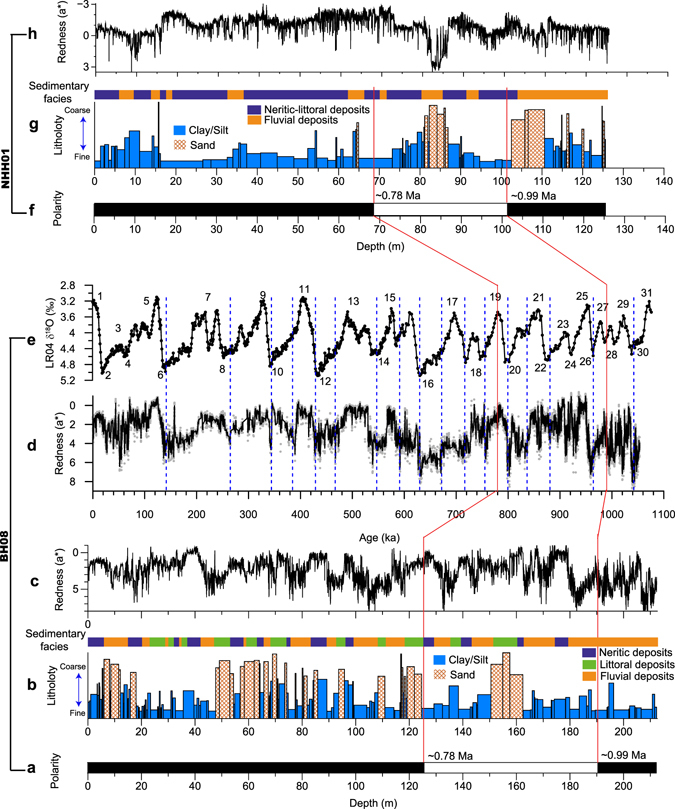
Integrated plot for cores BH08 and NHH01. (**a**) Magnetic polarity^17^. (**b**) Lithology and associated sedimentary facies^18^. Sediment color reflectance (redness; a*)^17^ at (**c**) depth scale and (**d**) age scale after astronomical tuning (5-point average smoothing) for core BH08. (**e**) Marine oxygen isotope record (LR04 stack)^68^ labeled the marine isotope stages with numbers. (**f**) Magnetic polarity, (**g**) Lithology and associated sedimentary facies, (**h**) Sediment color reflectance for core NHH01^16^. Red solid lines in all panels represent the boundary of Brunhes/Matuyama (~780 ka) and Jaramillo top (~990 ka). The blue dashed lines in panels (**d**) and (**e**) denote the age control points after tuning.

In this study, we therefore select two long cores from the Bohai Sea (core BH08; 212.4 m, 119.99°E, 38.28°N; Fig. 1b) and southern Yellow Sea (core NHH01; 125.6 m, 123.22°E, 35.22°N; Fig. 1b), respectively, which were dated back to the last ~1.1 Myr by magnetostratigraphic method (Fig. 2)^16, 17^. The astronomical tuning applied for the core BH08^17^ enabled the establishment of high-resolution chronology at the orbital timescale, based on the high-resolution parameter of sediment color (redness, a*; Fig. 2c and d) as a climate indicator on glacial-interglacial timescale. Core BH08 from the Bohai Sea is characterized by the alternations of marine and terrestrial deposits (Fig. 2b) over the last ~1 Myr with littoral-neritic settings corresponding to the interglacial high sea-level stands^17, 18^. Terrestrial deposits are the consequence of the fluvial setting, and the whole terrestrial sequence is dominated by fine-grained floodplain deposits^18^ (Fig. 2b). For the southern Yellow Sea core NHH01, marine-related deposits dominate in the upper ~105 m of the core and was dated to be ~990 ka (Fig. 2f), while the lower part was interpreted to be dominated by fluvial settings^16^ (Fig. 2g). The fluvial deposits in the lower part of the core NNH01 were characterized by several fluvial cycles with the dominance of floodplain deposits (Fig. 2g).

Clay minerals in marine sediments have been widely used to infer changes in the fine-grained sediment provenance, as well as the climatic condition in the source region^19–21^. Here we conducted a high-resolution measurement of clay minerals, aided by rare earth elements in these two cores to constrain the last ~1.1 Myr of sediment source to the study area. Our results show a climate-driven shift in sediment source from small, proximal mountain rivers-dominance to the Yellow River-dominance at ~880 ka, and suggest that the persistent effects of the Yellow River on Chinese marginal seas must have occurred at least ~880 ka ago.

## Results

**Figure 3:**
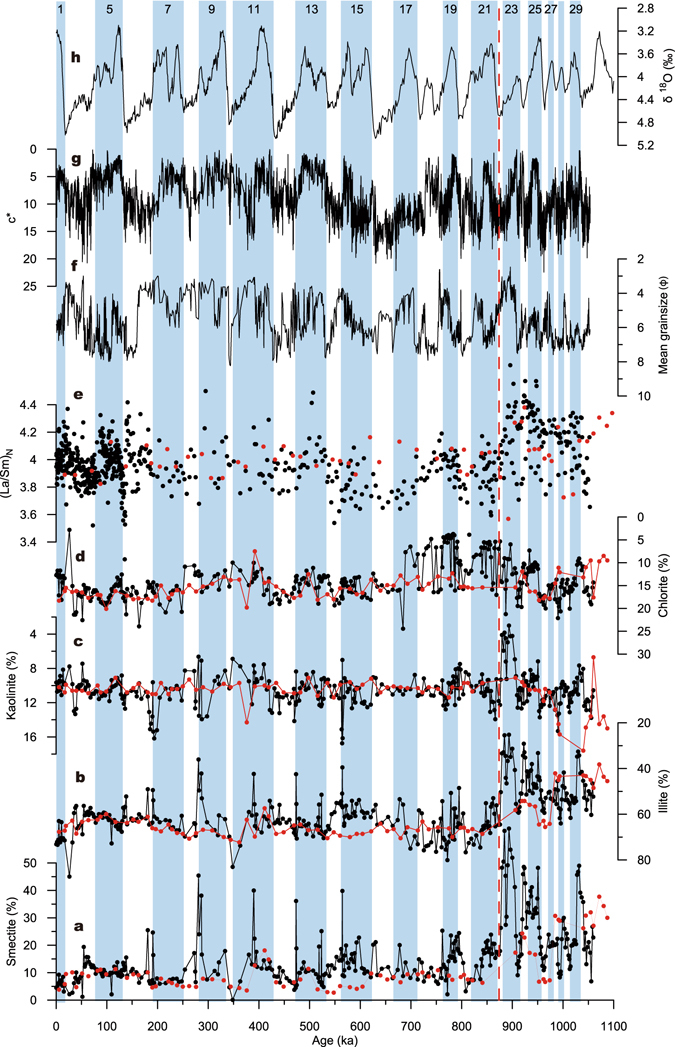
Temporal variations of clay minerals and La/Sm ratio in cores BH08 (black dots) and NHH01 (red dots) compared with marine oxygen isotope record (LR04). (**a**) Smectite. (**b**) Illite. (**c**) Kaolinite. (**d**) Chlorite. (**e**) Chondrite-normalized La/Sm ratio. (**f**) Mean grainsize. (**g**) Sediment color reflectance (c*, an indicator of transgression-regression cycles)^18^ of core BH08. (**h**) Stacked marine oxygen isotope record (LR04)^68^. The vertical shaded areas labeled with odd numbers represent the interglacial periods. The vertical dashed line denotes the age boundary of ~880 ka.

The clay mineral assemblage of core BH08 from the Bohai Sea is dominated by illite (average: ~59%), while smectite (average: ~16%), chlorite (average: ~14%) and kaolinite (average: ~10%) are less abundant. In core NHH01 from the southern Yellow Sea, illite, smectite, chlorite and kaolinite show averages (63%, 11%, 15% and 11%, respectively) similar to that of these clay minerals in core BH08 (Fig. 3). A prominent feature in clay mineral assemblages of core BH08 is that the relative content of illite is much higher, whereas the relative content of smectite is much lower in the upper ~150 m of core BH08 (Supplementary Fig. 1b and d). Furthermore, the content of smectite was much higher while illite was lower in littoral deposits than that of neritic and fluvial deposits between ~50 and 100 m of the core (Supplementary Fig. 1b and d). For core NHH01, a similar prominent change occurred at the depth of ~80 m (Supplementary Fig. 2b and d).

Based on the astronomically-tuned chronology^17^, the prominent change in illite and smectite at ~150 m in core BH08 (Supplementary Fig. 1b and d) corresponds to ~880 ka BP (Fig. 3a and b). A closer look of the clay mineral data of core BH08 reveals glacial-interglacial variations over the last ~1.1 Myr (Fig. 3). In general, illite, chlorite and kaolinite show similar cyclic changes, with lower values during the interglacials but higher values during the glacials (Fig. 3b-d). In contrast, smectite displays an opposite trend with higher values during the interglacial periods and lower values during the glacial periods (Fig. 3a). Interestingly, very high content of smectite and low content of illite were found at the end of interglacial periods of marine isotope stage (MIS) 7, 9, 11, 13 and 15 (Fig. 3a and b). Furthermore, these cyclic changes in illite and smectite are even more obvious with larger amplitudes prior to ~880 ka (Fig. 3). Although the sampling resolution of core NHH01 is relatively lower than core BH08, it also exhibits very similar variations for different clay minerals. Abrupt change in the clay mineral assemblage at ~880 ka, and very high (low) smectite (illite) during the end of MIS 7, 9, 11 and 13 (Fig. 3a and b) are also seen in chondrite-normalized La/Sm ratio, an indicator of enrichment of light REE over heavy REE, for both cores (Fig. 3e).

## Discussion

Export of clay minerals by rivers to the ocean is not only controlled by lithology of the provenance, but is also related to source rocks weathering intensity, which is closely linked to the climatic condition in the drainage basin^20, 21^. In addition, influence of hydrodynamic sorting on clay minerals due to the post-depositional alteration in coastal and marine environments has been also reported^19, 22, 23^. Provenance of sediments in the marine environment can thus be determined by comparing the proportion of clay minerals in marine sediments with that of the proportion of clay minerals in the surrounding fluvial sediments of potential sources, given that post-depositional alteration can be negligible.

Modern rivers flowing into the Bohai Sea include the Yellow River, Luan River, Hai River, Liao River, Liugu River, Daling River, Xiaoling River, Shuangtaizi River, Xiaoqing River and Wei River (Fig. 1b, Supplementary Table 1). The Yellow River originates from the northeastern part of the Tibetan Plateau in China, flows across the Chinese Loess Plateau (CLP), and finally discharges into the Bohai Sea. As its upper and middle courses flow through the CLP, the Yellow River is the most turbid river in the world and delivers a sediment load of 1.08 Gt/yr to the delta and the Bohai Sea^6^, representing about 90% of total sediments delivered to the Bohai Sea^15, 24^. In contrast to the Yellow River, other rivers including the Luan River, Hai River, Liao River, Liugu River, Daling River, Xiaoling River, Shuangtaizi River, Xiaoqing River and Wei River (Fig. 1b; Supplementary Table 1) flowing into the Bohai Sea, are shorter and originate from the northwestern mountain region of the Taihang-Yan Mountains and Shandong Peninsula around the Bohai Sea. The estimated contribution of sediments to the modern Bohai Sea by these rivers is less than 10%^24^.

The Yangtze River delivers a huge volume of water (920 km^3^) and sediment load (480 Mt) to the East China Sea annually^25^ (Fig. 1a). Although there is no large river from the Chinese continents directly flowing into the Yellow Sea, sediments transported by the Yellow River, the Yangtze River and small rivers around the Bohai Sea can potentially influence the sedimentary realm of the Yellow Sea, especially its southern part via the shelf current system^26^. There are also several small rivers (Fig. 1b; Yalujiang, Taedong, Chongchon, Han, Keum and Yeongsan Rivers) from Korean Peninsula flowing into the Yellow Sea. It has been estimated that about 5–25 Mt sediment delivered by Han, Keum and Yeongsan Rivers enters into the Yellow Sea annually^27^.

Sediments eroded from the CLP contribute ~90% of total sediment load to the Yellow River^15^. The particles and sediments of the Yellow River are characterized by high illite content, similar to that of the aeolian deposits in Northern China^28^. These deposits are detrital in nature and are believed to be derived from low-grade metamorphic rocks eroded from the source region^29^. In contrast to the dominance of illite in the Yellow River particulates, the short-length mountain rivers of Daling and Xiaoling around the Bohai Sea contain much higher smectite^30^ (Supplementary Table 2), which is attributed to weathering of mafic igneous rocks in the mountain catchment^31^ of these small rivers.

**Figure 4:**
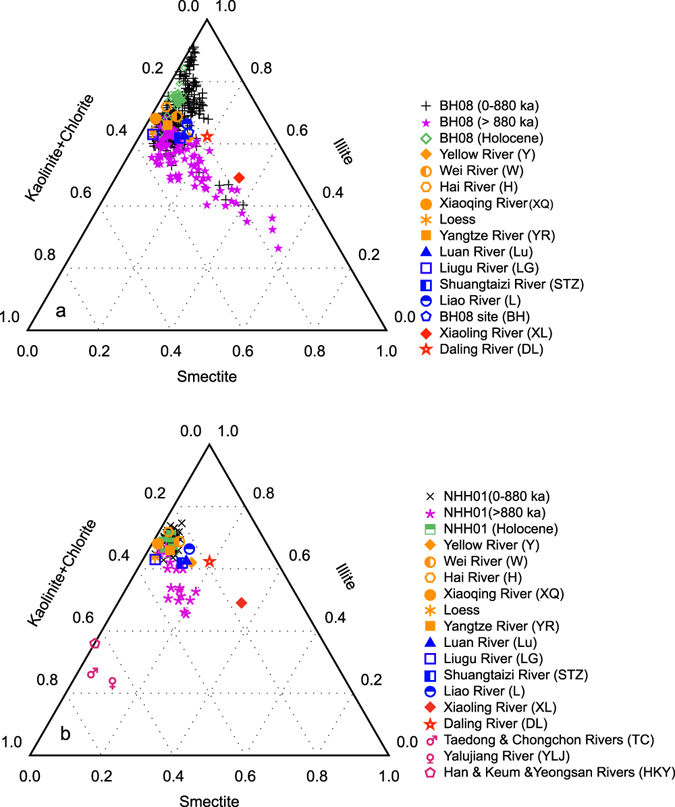
Ternary diagram of illite, kaolinite + chlorite and smectite for cores BH08 and NHH01 dividing at ~880 ka and comparison with potential provenance data. (**a**) Core BH08. (**b**) Core NHH01. The Holocene deposits for cores BH08 and NHH01 were determined by the AMS ^14^C results^16, 17^. Also shown are these clay minerals in modern river sediments^30, 69–72^, surface sediments^32^ around the BH08 site and loess samples^28^ for comparison.

To constrain the sediment source in cores BH08 and NHH01, illite, smectite and kaolinite + chlorite proportions of all investigated samples are plotted in a ternary diagram, along with modern river data of these minerals (Fig. 4). Sediments are plotted into two separate clusters for both cores (Fig. 4a and b). Sediments prior to ~880 ka plot close to the area represented by the clay mineral composition of the Daling and Xiaoling Rivers, whereas sediments younger than ~880 ka share the same area with the Yellow River, Yangtze River, aeolian deposits and other small rivers (Fig. 4a and b). Interestingly, clay minerals of surface sediments^32^ around the location of core BH08 and sediments accumulated during the entire Holocene interval also plot closer to that of the Yellow River, instead of the Daling and Xiaoling rivers (Fig. 4a). As the Yellow River has already flowed into the sea during the Holocene period^33^, this phenomenon thus indicates a provenance change at ~880 ka. Furthermore, this change seems to be characterized mainly by the transition from sediment discharge that dominated by the Daling and Xiaoling Rivers to that of the Yellow River dominance, consistent with clay minerals correlations (Supplementary Fig. 3) and their downcore variations (Fig. 3). The phenomenon that the data of Hai, Wei, Xiaoqing, Luan, Liao, Liugu and Shuangtaizi Rivers fall in the transition zone of these two clusters (Fig. 4a and b), possibly due either to the influence of the Yellow River signal because these rivers flow through the Yellow River drainage area in their lower reaches (e.g. Hai, Wei, Xiaoqing, Luan Rivers), or sediments does not originate from the mafic igneous mountains (e.g. Liao River). Clay mineral data of rivers from the Korean Peninsula plot far away from the area represented by the subsamples of core NHH01 (Fig. 4b), suggesting an insignificant contribution of clay minerals from these rivers to the core location. The overlap of Yangtze River data with samples after ~880 ka for both cores (Fig. 4) suggests the potential sediment contribution from the Yangtze River. However, much less sediment flux than the Yellow River (Supplementary Table 1) and the distance far away from the core sites could preclude the significant influence of the Yangtze River material in the study area.

**Figure 5:**
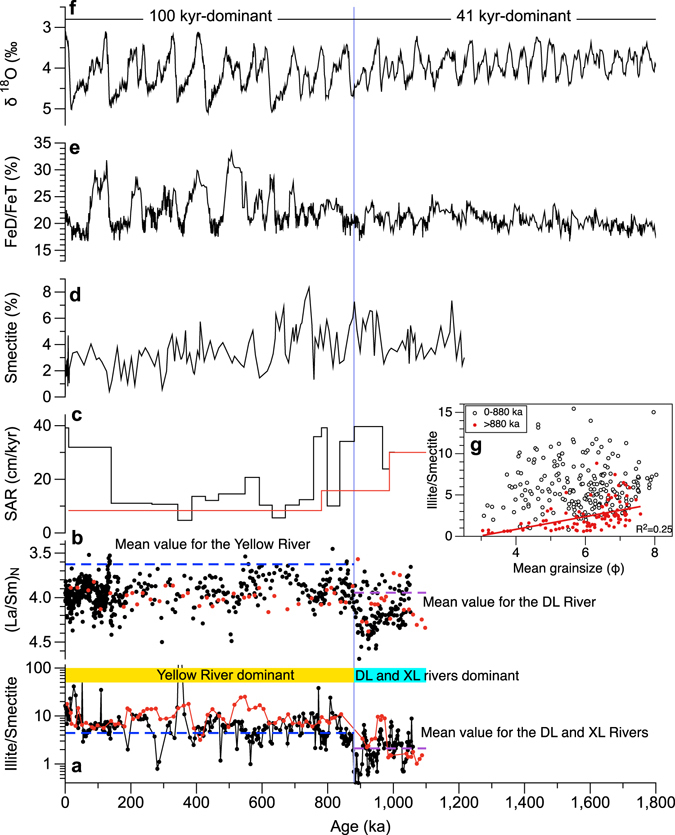
Comparison of proxies for cores BH08 (black) and NHH01 (red) with paleoclimate records. (**a**) Illite/smectite. (**b**) Chondrite-normalized La/Sm ratio. (**c**) Sedimentary accumulation rate (SAR) for cores BH08^17^ and NHH01^16^. (**d**) Smectite content for Lingtai section^40^. (**e**) Stacked FeD/FeT ratio from loess-soil sequence in northern China^56^. (**f**) Stacked marine oxygen isotope^68^. (**g**) Correlation between illite/smectite and mean grainsize for core BH08. Mean values of illite/smectite for the Yellow River^72^, Daling (DL) and Xiaoling (XL) rivers^30^ are plotted as horizontal lines in panel (**a**), and La/Sm for the Yellow River^35^ and Daling River^36^ in panel (**b**). Vertical line denotes the age boundary of ~880 ka.

In addition to the effect of provenance, which is regarded as the first order control on the clay mineral composition for both cores, climate can also modulate changes in the clay mineral assemblage^19, 20^. Illite and chlorite are indicators of weak hydrolysis in continental weathering and strong erosion under cold-arid climatic conditions^19^. However, kaolinite and smectite are products of intense chemical weathering and can be readily found in regions characterized by warm-humid climate with enhanced continental hydrolysis^19^. Glacial-interglacial patterns of clay minerals in cores BH08 and NHH01 (Fig. 3) suggest that climate may also exert a secondary control on the clay mineral variations at orbital timescales. Higher illite and chlorite contents during the glacial periods indicate cold-dry climatic conditions, while more smectite during the interglacial intervals suggests warm-humid conditions in the drainage area. The orbital variations in the illite/smectite ratio, a proxy for the relative proportion of the Yellow River to the Daling and Xiaoling Rivers, correlate well with the grain size prior to ~880 ka (Fig. 5a and g), with coarser (finer) fraction corresponding to lower (higher) illite/smectite ratios in the interglacials (glacials) (Figs 3 and 5). There is no such distinct climate-grain size link in sediments accumulated younger than ~880 ka (Fig. 5g), suggesting a more obvious climatic control on the production of clay minerals prior to ~880 ka when the sediment source was mostly from the proximal mountain rivers. On the other hand, after ~880 ka, large amounts of sediments were delivered from distant, multi-sources in the Yellow River drainage that might have complicated the clay mineral composition in the core sequence. The ratio of chondrite-normalized La/Sm ratio has been used to quantify the degree of light rare earth element (LREE) enrichment over heavy rare earth element (HREE)^34^. In addition, LREE primarily reside in clay minerals^34^ and thus increasing clay minerals content in sediments can also show an LREE enrichment. The inference obtained from the clay mineral was also supported by the La/Sm ratio which suggests a provenance change from light REE depleted to enriched at the same time (Figs 3e and 5b), as La/Sm ratio of the modern Yellow River sediments is lower (3.57)^35^ (Fig. 5b) than that of the Daling River (3.95)^36^ (Fig. 5b). Higher La/Sm ratio in sediments accumulated prior to 880 ka might be the effect of weathering of mafic igneous rocks in the catchments of the Daling and Xiaoling Rivers^37^.

It should be noted that the post-depositional alteration, such as frequent alternations of transgression and regression in the study area which involves the hydrodynamic sorting on clay minerals^19, 22, 23^, can potentially influence the composition of clay minerals. It has been suggested that smectite is prone to winnowing by oceanic currents due to the smallest size of all clay minerals^19^. However, very small variations in clay minerals between marine and fluvial deposits since ~880 ka and higher smectite in marine deposits suggest that the post-depositional alteration might be very limited, compared with the significant provenance change occurred at ~880 ka.

There are two additional causes for the absence of the Yellow River sedimentary signal in the Bohai Sea prior to ~880 ka: (i) the shift of the Yellow River course towards the southern Yellow Sea (Fig. 1), and (ii) the clay mineral composition of sediments transported by the Yellow River itself may have changed since that time. For instance, both sedimentary and historical records indicate that the Yellow River had changed its lower course several times in the past and emptied into the Yellow Sea^15, 38^. The most recent change had occurred at 1855 AD during when the river made a sharp turn northeastward and flowed across the North China plain before flowing into the Bohai Sea, whereas it wandered eastward to the southern Yellow Sea prior to 1855 AD^39^. However, we suggest that the change in clay mineral was not the consequence of Yellow River channel shifting, because the same provenance change was also recorded in core NNH01 investigated from the southern Yellow Sea (Fig. 3). As the aeolian materials are the major contributor of the Yellow River sediments to the marginal seas, the abrupt provenance change in CLP that might have led to the difference in the clay mineral in our core sequence. However, clay mineralogical study of a Quaternary loess sequence from Lingtai section of CLP^40^ suggested that there was no abrupt change in illite and smectite contents at ~880 ka (Fig. 5d). Meanwhile, the smectite for the Lingtai section is quite low during the last ~1.2 Myr with a mean content of <~7%^40^ (Fig. 5d), indicating that change in the formation of clay mineral likely insufficient to provide so much of smectite, as recorded in the BH08 sequence prior to ~880 ka.

Previous evidence on the origin and drainage history of the Yellow River relies on the studies of fluvial terrace in the Yellow River drainage area. Integrated geological evidence suggests that the Yellow River drained eastward directly to the ocean as early as in the Eocene, and its square bend developed in the early Pliocene^12^. Based on the stratigraphic, geochronological and geomorphic data, Craddock *et al*.^9^ suggested that the appearance of the Yellow River and headward basin integration had occurred during the period between ~1.8 and ~0.5 Myr^9^. The Sanmen Gorge is the last gorge that Yellow River traversing the middle and lower reaches to the sea (Fig. 1a). The timing of cut-through this gorge is therefore widely regarded as the final integration of the Yellow River. Based on the paleomagnetic dating and loess-soil correlation of fluvial terrace at the entrance of the Sanmen Gorge, Pan *et al*.^41^ suggested that the Yellow River cut through the Sanmen Gorge into the Chinese seas no later than 1.165 Myr^41^. Kong *et al*.^11^ also reported a similar cutting-through timing of 1.3–1.4 Myr using cosmogenic ^10^Be/^26^Al dating of fluvial terraces and buried sedimentary sequence, along with provenance discrimination using detrital zircon U-Pb age distribution^11^. Much younger timing, however, for the Yellow River cutting-through the Sanmen Gorge was also reported. For example, based on the abrupt increase in both coarse fraction and accumulation rates of the aeolian deposits in the southern side of the Sanmen Gorge at ~150 ka, Jiang *et al*.^10^ attributed this change to the down-cutting of the Sanmen Gorge by the Yellow River^10^ (Fig. 1a). This age was later re-interpreted to be ~200–250 ka (MIS 7)^42^, based on the close spatial correlation among different loess sections using magnetic susceptibility.

Records of clay minerals and La/Sm ratio suggest that the Yellow River began to influence the Bohai and Yellow Seas at least ~880 ka ago and persist until today (Fig. 5). This time is younger than earlier results from the geomorphological studies in the Yellow River drainage area^9, 11, 12, 41^, but much older than the results reported by Jiang *et al*.^10^ and Zheng *et al*.^42^. Evidence from sedimentology and monazite age spectrum suggested that the Yangtze River flowing into the East China Sea was at around the Early Pleistocene^43^. However, a recent study with a combination of sedimentology, ^40^Ar/^39^Ar ages from basalts interbedded with fluvial sediments from the lower reaches of the Yangtze River, and U–Pb ages of detrital zircon from sand grains suggested a much older age for the Yangtze River and implied that the modern drainage systems in eastern China had already formed at least by the early Miocene^44^. As the Yellow River shares its birth place with the Yangtze River (red square in Fig. 1a), it is most likely that the Yellow River might have formed as early as at that time. This speculation is partly supported by geological evidence, which reveals that the Yellow River formed in the Eocene, but with a more direct path eastward into the Bohai Sea^12^. The subsequent evolution, including the formation of the square bend and the fluvial terrace, might be just the local adjustments of the Yellow River and its tributaries. Limited by the core length, our data do not allow us to date the initiation age of the Yellow River, but we infer that the river has influenced the Chinese marginal seas at least ~880 ka ago and it persisted until the present time.

River development in general follows climatic evolution, and phases of morphological instability and thus erosion have been generally identified during the climatic transitions^45, 46^. On geological timescales, the geomorphological effect of river activity is best expressed at times of glacial-interglacial climatic change^45^. In the glacial periods, though relatively large amounts of sediment were supplied to the rivers, most sediment could be transported only during the period of interglacials when river had high transport-capacity^47^ due to strengthened monsoonal precipitation. During the Quaternary, global climate has changed into cold and dry, and was characterized by alternations of glacial and interglacial^48^. However, the glacial-interglacial pattern, in terms of both amplitude and duration had increased across the mid-Pleistocene transition (MPT), the interval characterized by a change from a dominant 41-kyr to 100-kyr ice-age cyclicity at around 0.9 Myr^49, 50^ (Fig. 5f).

Since many previously published evidence points to older than 880 ka for the final integration of the Yellow River^9, 11, 12^, we suggest that the clay mineral changes expressed by increased smectite but decreased illite throughout the sequence can be a consequence of fluvial response to climate change, which led to the shift of dominant-provenance. Prior to ~880 ka, warmer climate^51^ and smaller glacial-interglacial variations both in climate and sea-level^52, 53^ (Fig. 5f) must have facilitated the maintenance of the constancy of river system. This is partly supported by finer grainsize in core BH08 with smaller fluctuations (Fig. 3f). Hence, one would expect that most of the sediments delivered by the Yellow River were likely trapped in lower reaches of the river^54^, and only a small portion of sediments was transported to the sea. This inference is supported by a recent sediment budget estimate of the Yellow River that indicated the trapping of about 70% of the materials carried by the Yellow River in the North China Plain^24^. Nonetheless, the Daling and Xiaoling Rivers, as well as other mountain rivers around the Bohai Sea are much closer to the core location and therefore eroded sediments can be easily transported to the core site. This can explain the high smectite in the sediments accumulated prior to ~880 ka (Fig. 3a), especially for the interglacials. However, the grainsize is finer than that for the last ~880 ka, possibly due to less monsoon precipitation (Fig. 5e) and/or smaller glacial-interglacial variations in terms of both climate and sea-level changes (Fig. 5f).

The sediment delivery has likely been changed after ~880 ka under the situation of large amplitudes and long duration of glacial-interglacial cycles^48^. Under increased glacial-interglacial contrasts of climate condition, the intensity of summer monsoon displays large variations since that time, as suggested by micromorphological investigations on the loess deposits in the northern China^55^ and increased fluctuations in FeD/FeT, an index of summer monsoon intensity^56^ (Fig. 5e). Meanwhile, increased fluctuations in sea-level after ~880 ka tend to have promoted the instability of the river system, which caused frequent aggradation and incision in the interglacial-glacial cycles^57^. Thus, large amounts of materials eroded in the catchment area in the glacials could be transported much lower reaches of the river system under high precipitation-induced discharge period. The poor vegetation cover in the northern China^58^ as a result of cooler climate condition^51^ may further intensify the delivery of the Yellow River sediments into the ocean. It should be noted that the influence of the mountain-drained rivers might persist throughout the last ~1 Myr. The decreased contribution of the Daling and Xiaoling Rivers since ~880 ka is most likely due to the dilution of the Yellow River, which transported huge amounts of materials from its drainage basin.

Nevertheless, on several occasions extremely high smectite can be found in littoral deposits over the last ~880 ka (Fig. 3a), mainly in MIS 7, 9, 11, 13 and 15. These smectite peaks confirm the strengthened monsoon precipitation during these periods^59^, enabling the transport of eroded materials from the proximal mountain regions to the study area. The coarse-grained sediments with higher sand fraction (>80%; Fig. 3f) in these intervals partly support this idea, as the texture of the Yellow River sediments are mainly dominated by the fine-grained silt fraction.

In addition to the climate change, a number of studies with various lines of evidence revealed a rapid uplift of the Tibetan Plateau in the mid-Pleistocene at about 1.2–0.8 Myr^60–62^. Studies implicate that tectonic factor may exert a major control on the integration of the isolated basin in the Yellow River drainage area and its final flowing into the ocean^12^. A recent study on the aspects of magnetostratigraphy and sedimentology from the Bohai Sea suggested a local tectonic control of environmental transition from lacustrine to marine setting that occurred at ~1 Ma^63^. However, the glacial-interglacial pattern of clay mineral assemblage in core BH08 might preclude the tectonic factor and implies that provenance change is climate-controlled. Furthermore, two- to three-fold decreased sedimentation rate at ~800–900 ka in cores NHH01 and BH08^16, 17^ (Fig. 5c) provide no support for the major tectonic control on this provenance change. Taken together, our results provide the robust evidence that the persistent influence of the Yellow River on the Chinese marginal seas initiated at least ~880 ka ago.

## Methods

### Color reflectance measurements

The color reflectance of sediments for core NHH01 was measured using a Minolta CM-2002 hand-held spectrometer at ~1 cm interval. The results were expressed as a three-dimensional color system L*, a* and b*. L* describes the lightness between black (0) and white (100), while a* and b* denote the red (positive values)–green (negative values) and yellow (positive values)–blue (negative values) chromaticity, respectively.

### X-ray diffraction (XRD) analysis

For the present study, 429 subsamples (348 subsamples from core BH08 and 81 subsamples from core NHH01) were selected at ~0.5–2 m intervals for clay mineral analysis. Prior to the clay mineral measurement, all samples were treated with 30% H_2_O_2_ and 0.5 M HCl for 24 h to remove organic matter and carbonate, respectively. Clay-size particles (<2 µm) were separated following the Stokes’ law and concentrated by centrifuging. Clay minerals were measured by X-ray Diffraction (XRD) with CuKα radiation using a D/Max 2500PC diffractometer at a voltage of 40 kV and an intensity of 100 mA. Identification of clay minerals was made for smectite (1.7 nm), illite (1 nm) and kaolinite + chlorite (0.7 nm) on the glycolated curve. Kaolinite and chlorite were separated by relative proportions according to their high ratios of 0.357/0.354 nm. For the semi-quantitative analysis of clay minerals, we followed the method of Biscaye (1965)^64^.

### Rare earth elements measurements

Rare earth elements (REEs) are characterized by strong partitioning into the particulate phase, coherent behavior during weathering, erosion, and fluvial transportation, and high resistance to chemical mobilization^65^. Thus, REEs have been widely used as tracers for determining sediment sources^66^. In this study, a total of 715 subsamples (656 from core BH08 and 59 subsamples from core NHH01) were selected at ~0.1–2 m intervals for REEs analysis, following the method described in Zou *et al*.^67^. Briefly, all sediment samples were first digested with HNO_3_-HF (1:1) in closed Teflon beakers, and then analyzed with ICP-MS for REEs analysis. The entire experimental process was under quality control by a blank experiment, GSD-9 standard material and replicate measurements. The relative standard deviation for REEs analysis is less than 5%.

## Electronic supplementary material

Supplementary Info

## References

[1] Brookfield ME (1998). The evolution of the great river systems of southern Asia during the Cenozoic India-Asia collision: rivers draining southwards. Geomorphology.

[2] Bianchi, T. S., Allison, M. A. & Cai, W. J. *Biogeochemical Dynamics at Major River-coastal interfaces*. Cambridge University Press (2014).

[3] Homoky WB, John SG, Conway TM, Mills RA (2013). Distinct iron isotopic signatures and supply from marine sediment dissolution. Nat. Commun..

[4] Johnson KS, Chavez FP, Friederich GE (1999). Continental-shelf sediment as a primary source of iron for coastal phytoplankton. Nature.

[5] Chiocci, F. L. & Chivas, A. R. Continental shelves of the world: their evolution during the last Glacio-Eustatic cycle. The Geological Society London (2014).

[6] Milliman JD, Meade RH (1983). World-wide delivery of river sediment to the oceans. J. Geol..

[7] Raymo ME, Ruddiaman WF (1992). Tectonic forcing of late Cenozoic climate. Nature.

[8] Willenbring JK, Blanckenburg FV (2010). Long-term stability of global erosion rates and weathering during late-Cenozoic cooling. Nature.

[9] Craddock WH (2010). Rapid fluvial incision along the Yellow River during headward basin integration. Nat. Geosci..

[10] Jiang F, Fu J, Wang S, Sun D, Zhao Z (2007). Formation of the Yellow River, inferred from loess–palaeosol sequence in Mangshan and lacustrine sediments in Sanmen Gorge, China. Quat. Int..

[11] Kong P, Jia J, Zheng Y (2014). Time constraints for the Yellow River traversing the Sanmen Gorge. Geochem. Geophys. Geosyst..

[12] Lin A, Yang Z, Sun Z, Yang T (2001). How and when did the Yellow River develop its square bend?. Geology.

[13] Pan B (2012). The approximate age of the planation surface and the incision of the Yellow River. Palaeogeogr. Palaeoclimatol. Palaeoecol..

[14] Ren ME, Shi YL (1986). Sediment discharge of the Yellow River (China) and its effect on the sedimentation of the Bohai and the Yellow Sea. Cont. Shelf Res..

[15] Yang SY, Cai JG, Li CX, Deng B (2001). New discussion about the run-through time of the Yellow River (in Chinese). Mar. Geol. Quat. Geol..

[16] Liu J (2014). Magnetostratigraphy of a greigite‐bearing core from the South Yellow Sea: Implications for remagnetization and sedimentation. J. Geophys. Res. Solid Earth.

[17] Yao ZQ (2014). Paleomagnetic and astronomical dating of sediment core BH08 from the Bohai Sea, China: Implications for glacial–interglacial sedimentation. Palaeogeogr. Palaeoclimatol. Palaeoecol..

[18] Shi XF (2016). Sedimentary architecture of the Bohai Sea China over the last 1 Ma and implications for sea-level changes. Earth Planet. Sci. Lett..

[19] Chamley, H. *Clay Sedimentology*. Springer-Verlag (1989).

[20] Thiry M (2000). Palaeoclimatic interpretation of clay minerals in marine deposits: an outlook from the continental origin. Earth Sci. Rev..

[21] Liu Z (2016). Source-to-sink transport processes of fluvial sediments in the South China Sea. Earth Sci. Rev..

[22] Dou Y (2010). Clay mineral evolution in the central Okinawa Trough since 28ka: Implications for sediment provenance and paleoenvironmental change. Palaeogeogr. Palaeoclimatol. Palaeoecol..

[23] Wang Q, Yang SY (2013). Clay mineralogy indicates the Holocene monsoon climate in the Changjiang (Yangtze River) Catchment, China. Appl. Clay Sci..

[24] Cheng TW, Zhao CN (1985). Runoff volumes and sediment discharges of large rivers in China and their influence on the coastal zone. Acta Oceanolog. Sin..

[25] Milliman JD, Syvitski JP (1992). Geomorphic/tectonic control of sediment discharge to the ocean: the importance of small mountainous rivers. J. Geol..

[26] Ren ME (2015). Sediment discharge of the Yellow River, China: past, present and future—a synthesis. Acta Oceanolog. Sin..

[27] Lim DI, Choi JY, Jung HS, Rho KC, Ahn KS (2007). Recent sediment accumulation and origin of shelf mud deposits in the Yellow and East China Seas. Prog. Oceanogr..

[28] Shi Y, Dai X, Song Z, Zhang W, Wang L (2005). Characteristics of clay mineral assemblages and their spatial distribution of Chinese Loess in different climatic zones. Acta Sedimentol. Sin..

[29] Ji J, Chen J, Lu H (1999). Origin of illite in the loess from the Luochuan area, Loess Plateau, Central China. Clay Miner..

[30] Dou Y (2014). Clay mineral distributions in surface sediments of the Liaodong Bay, Bohai Sea and surrounding river sediments: Sources and transport patterns. Cont. Shelf Res..

[31] Ma, L. *et al*. *Geological Atlas of China*. Geological Publishing House, Beijing (2002).

[32] Shi, X. F. Chinese Marginal Seas: Marine Bottom Sediments. China Ocean press (2012).

[33] Saito Y, Yang Z, Hori K (2001). The Huanghe (Yellow River) and Changjiang (Yangtze River) deltas: a review on their characteristics, evolution and sediment discharge during the Holocene. Geomorphology.

[34] He MY (2015). Geochemistry of fine-grained sediments in the Yangtze River and the implications for provenance and chemical weathering in East Asia. Prog. Earth Planet. Sci..

[35] Xu ZK, Lim D, Choi J, Yang SY, Jung H (2009). Rare earth elements in bottom sediments of major rivers around the Yellow Sea: implications for sediment provenance. Geo-Mar. Lett..

[36] Zhou GH, Sun BB, Liu ZY, Wei HL, Zeng DM (2012). Geochemical feature of rare earth elements in major rivers of Eastern China (in Chinese with English abstract). Geoscience.

[37] Yang, Z. J., Jin, Z. & Zhang, J. Y. *Liaoning Regional Geology* (*in Chinese*). Geological Publishing House (1989).

[38] Yu LS (2002). The Huanghe (Yellow) River: a review of its development, characteristics, and future management issues. Cont. Shelf Res..

[39] Yang W (2009). Relocation of the Yellow River as revealed by sedimentary isotopic and elemental signals in the East China Sea. Mar. Pollut. Bull..

[40] Gylesjö S, Arnold E (2006). Clay mineralogy of a red clay–loess sequence from Lingtai, the Chinese Loess Plateau. Global Planet. Change.

[41] Pan B (2005). Paleomagnetic dating of the topmost terrace in Kouma, Henan and its indication to the Yellow River’s running through Sanmen Gorges. Chin. Sci. Bull..

[42] Zheng H, Huang X, Ji J, Liu R, Zeng Q (2007). Ultra-high rates of loess sedimentation at Zhengzhou since Stage 7: Implication for the Yellow River erosion of the Sanmen Gorge. Geomorphology.

[43] Fan D (2005). Monazite age spectra in the Late Cenozoic strata of the Changjiang delta and its implication on the Changjiang run-through time. Sci. China (D).

[44] Zheng H (2013). Pre-Miocene birth of the Yangtze River. Proc. Nati. Acad. Sci.

[45] Vandenberghe J (1995). Timescales, climate and river development. Quat. Sci. Rev..

[46] Vandenberghe J (2003). Climate forcing of fluvial system development: an evolution of ideas. Quat. Sci. Rev..

[47] Vandenberghe J (2002). The relation between climate and river processes, landforms and deposits during the Quaternary. Quat. Int..

[48] Shackleton NJ (1987). Oxygen isotopes, ice volume and sea level. Quat. Sci. Rev..

[49] Clark PU (2006). The middle Pleistocene transition: characteristics, mechanisms, and implications for long-term changes in atmospheric pCO2. Quat. Sci. Rev..

[50] Ruddiman WF, Raymo ME, Martinson DG, Clement BM, Backman J (1989). Pleistocene evolution: northern hemisphere ice sheets and north Atlantic ocean. Paleoceanography.

[51] Zachos JC, Pagani M, Sloan L, Thomas E, Billups K (2001). Trends, Rhythms, and Aberrations in Global Climate 65 Ma to Present. Science.

[52] Rohling EJ (2014). Sea-level and deep-sea-temperature variability over the past 5.3 million years. Nature.

[53] Grant, K. *et al*. Sea-level variability over five glacial cycles. *Nat*. *Commun*. **5** (2014). 10.1038/ncomms6076 25254503

[54] Nie JS (2015). Loess Plateau storage of Northeastern Tibetan Plateau-derived Yellow River sediment. Nat. Commun..

[55] Guo ZT, Liu DS, Fedoroff N, An ZS (1993). Shift of the monsoon intensity on the loess plateau at ca 0.85 Ma. Chin. Sci. Bull..

[56] Guo ZT (2000). Summer monsoon variations over the last 1.2 Ma from the weathering of loess-soil sequences in China. Geophys. Res. Lett..

[57] Zhuo H (2015). Contrasting fluvial styles across the mid-Pleistocene climate transition in the northern shelf of the South China Sea: Evidence from 3D seismic data. Quat. Sci. Rev..

[58] Fan, S. X. *et al*. Palaeovegetation and environmental evolution in Hengshui district of Hebei province since 3.5 Ma BP (in Chinese). *Geoscience*, 75–81 (2009).

[59] Liu TS, Ding ZL (1998). Chinese Loess and the paleomonsoon. Annu. Rev. Earth Planet. Sci..

[60] Cui Z (1998). On Kunlun-Yellow River tectonic movement. Sci. China (D).

[61] Fang X (2005). Late Cenozoic deformation and uplift of the NE Tibetan Plateau: Evidence from high-resolution magnetostratigraphy of the Guide Basin, Qinghai Province, China. Geol. Soc. Am. Bull.

[62] Liu D (2010). Stratigraphic and paleomagnetic evidence of mid-Pleistocene rapid deformation and uplift of the NE Tibetan Plateau. Tectonophysics.

[63] Yi L (2016). Plio-Pleistocene evolution of Bohai Basin (East Asia): demise of Bohai Paleolake and transition to marine environment. Sci. Rep.

[64] Biscaye PE (1965). Mineralogy and sedimentation of recent deep-sea clay in the Atlantic Ocean and adjacent seas and oceans. Geol. Soc. Am. Bull.

[65] McLennan SM (1989). Rare earth elements in sedimentary rocks; influence of provenance and sedimentary processes. Rev. Mineral. Geochem..

[66] Yang SY, Jung HS, Choi MS, Li CX (2002). The rare earth element compositions of the Changjiang (Yangtze) and Huanghe (Yellow) river sediments. Earth Planet. Sci. Lett..

[67] Zou JJ (2015). Evidence of sea ice-driven terrigenous detritus accumulation and deep ventilation changes in the southern Okhotsk Sea during the last 180 ka. J. Asian Earth Sci..

[68] Lisiecki, L. E. & Raymo, M. E. A Pliocene-Pleistocene stack of 57 globally distributed benthic δ^18^O records. *Palaeoceanography* **20**, PA1003, doi:1010.1029/2004PA001071 (2005).

[69] Li J (2014). Provenance variations in the Holocene deposits from the southern Yellow Sea: Clay mineralogy evidence. Cont. Shelf Res..

[70] Li Y, Li AC, Huang P, Xu FJ, Zheng XF (2014). Clay minerals in surface sediment of the north Yellow Sea and their implication to provenance and transportation. Cont. Shelf Res..

[71] Park YA, Khim BK (1992). Origin and dispersal of recent clay minerals in the Yellow Sea. Mar. Geol..

[72] Yang SY, Jung HS, Lim DI, Li CX (2003). A review on the provenance discrimination of sediments in the Yellow Sea. Earth Sci. Rev..

