# Supplementary material for: Persistent effects of the Yellow River on the Chinese marginal seas began at least ~880 ka ago
Source: Sci Rep. 2017 Jun 6;7:2827. doi: 10.1038/s41598-017-03140-x (PMC5460111; doi:10.1038/s41598-017-03140-x)
Supplement: Supplementary file 1 — Supplementary Info [file 41598_2017_3140_MOESM1_ESM.pdf]

Persistent effects of the Yellow River on the Chinese marginal seas began at least ~880 ka ago

Zhengquan Yao, Xuefa Shi, Shuqing Qiao, Qingsong Liu, Selvaraj Kandasamy, Jianxing Liu, Yanguang Liu, Jihua Liu, Xisheng Fang, Jingjing Gao, Yanguang Dou

Supplementary Information:

Supplementary Table 1

Supplementary Table 2

Supplementary Figure 1

Supplementary Figure 2

Supplementary Figure 3

Supplementary Table 1

General information of major rivers flowing into the Bohai, Yellow and East China Seas

River	Length (km)	Drainage area (10^4 km^2)	Water discharge (10^8 m^3/yr)	Sediment flux (10^6 t/yr)	Statistical year	Reference
Yellow River	5464	75	350	880	1950-1998	1
Liao River	1396	12.76	31.9	15.4	1950-2010	2
Luan River	877	4.49	19.8	10.8	1950-2012	3
Hai River	1036	5.22	15.55	17.4	1950-2010	1
Daling River	435	2.32	20.6	17.7	1956-2000	4
Wei River	242	0.64	14.5	0.92	-	5
Xiaoqing River	233	1.05	8.78	0.37	-	5
Xiaoling River	206	0.55	3.98	2.24	-	4
Liugu River	158	0.31	6.84	0.98	-	4
Shuangtaizi River	116	0.25	27.5	1.73	-	5
Yangtze River	6390	180	8964	390	1950-2010	6

Supplementary Table 2

Average values of different clay minerals for major river sediments and loess deposits. For comparison, clay mineral contents of core sediments in different time intervals are also included.

River/Sediments	Abbreviation	Sample No.	Illite (%)	Chlorite (%)	Kaolinite (%)	Smectite (%)	Reference
Liao River	L	3	66	9	13	11	7*
Luan River	Lu	5	63	12	13	12	7*
Daling River	DL	2	62	5	14	19	7*
Xiaoling River	XL	3	49	7	10	34	7*
Shuangtaizi River	STZ	3	62	12	14	11	7*
Liugu River	LG	3	63	15	18	3	7*
Hai River	H	2	72	15	10	3	7*
Wei River	W	2	69	10	14	7	7*
Xiaoqing River	XQ	1	68	15	15	2	7*
Yellow River	Y	8	62	16	10	12	8
Yangtze River	CJ	8	66	12	16	6	8
Taedong & Chongchon Rivers	TC	21	70	14	12	4	9
Yalujiang River	YLJ	10	65	21	3	11	10
Han & Keum & Yeongsan Rivers	HKY	/	64	18	18	0.1	8,11
Loess	Loess	21	67	20	10	3	12
Surface samples around core BH08 site	BH	5	63	14	10	13	13
BH08 deposits younger than 880 ka	BH08<880	221	63	14	10	12	This study
BH08 deposits older than 880 ka	BH08>880	97	48	15	10	27	this study
Holocene deposits in core BH08	BH08 (Holocene)	13	70	14	10	6	this study
NHH01 deposits younger than 880 ka	NHH01<880	62	66	16	10	8	this study
NHH01 deposits older than 880 ka	NHH01>880	19	51	14	12	23	this study
Holocene deposits in core NHH01	NHH01 (Holocene)	6	66	17	10	7	this study

* Recalculated on the original data following the same method for studied cores

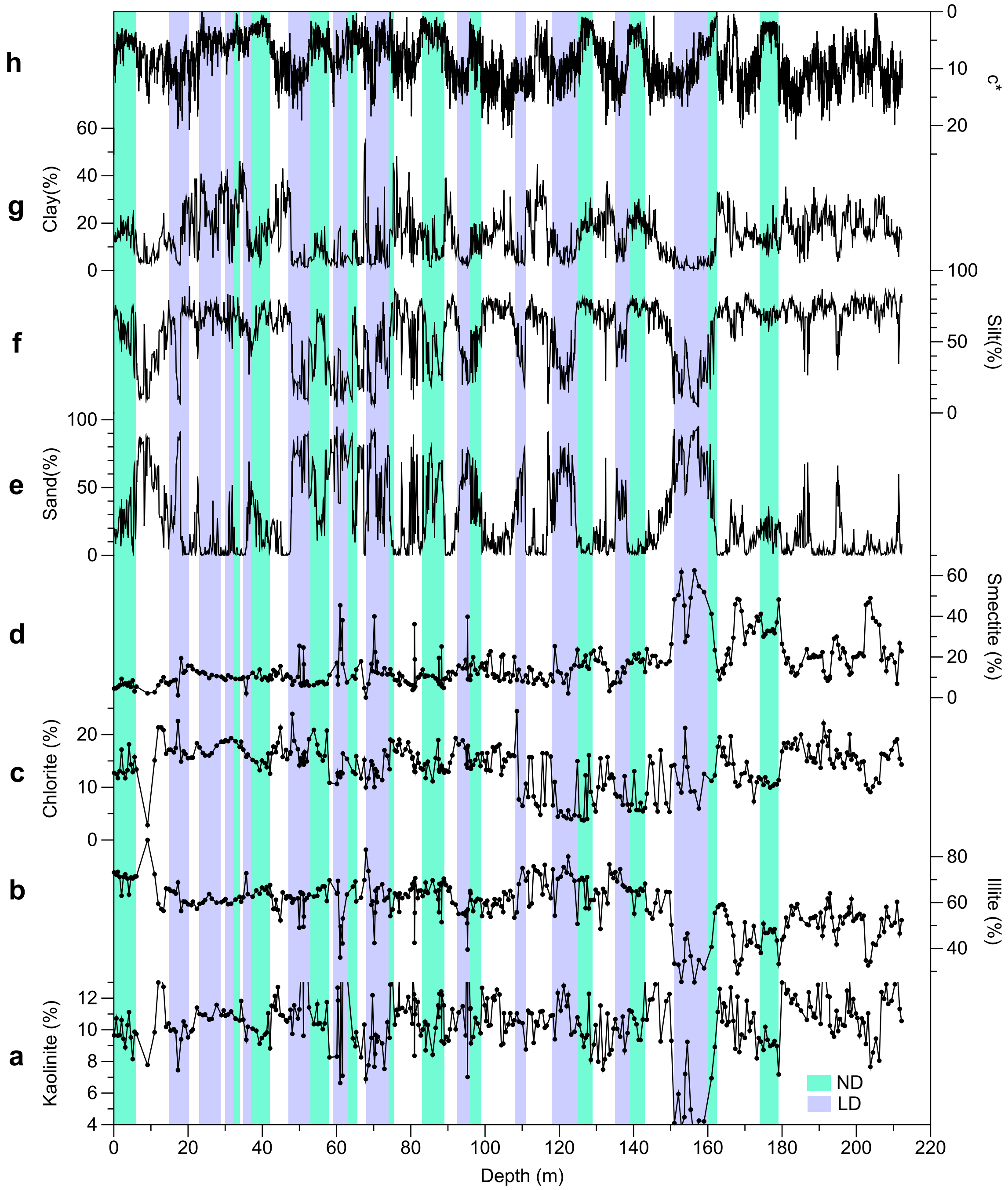

Supplementary Figure 1. Clay mineralogical and sedimentological parameters against depth in core BH08. (a) Kaolinite. (b) Illite. (c) Chlorite. (d) Smectite. (e) Contents of sand[14], (f) silt[15] and (g) clay[14]. (h) Sediment color reflectance (c*, an indicator of transgression-regression cycles)[15]. The green and purple shades represent neritic deposits (ND) and littoral deposits (LD), respectively, while the blank area represents fluvial deposits in core BH08.

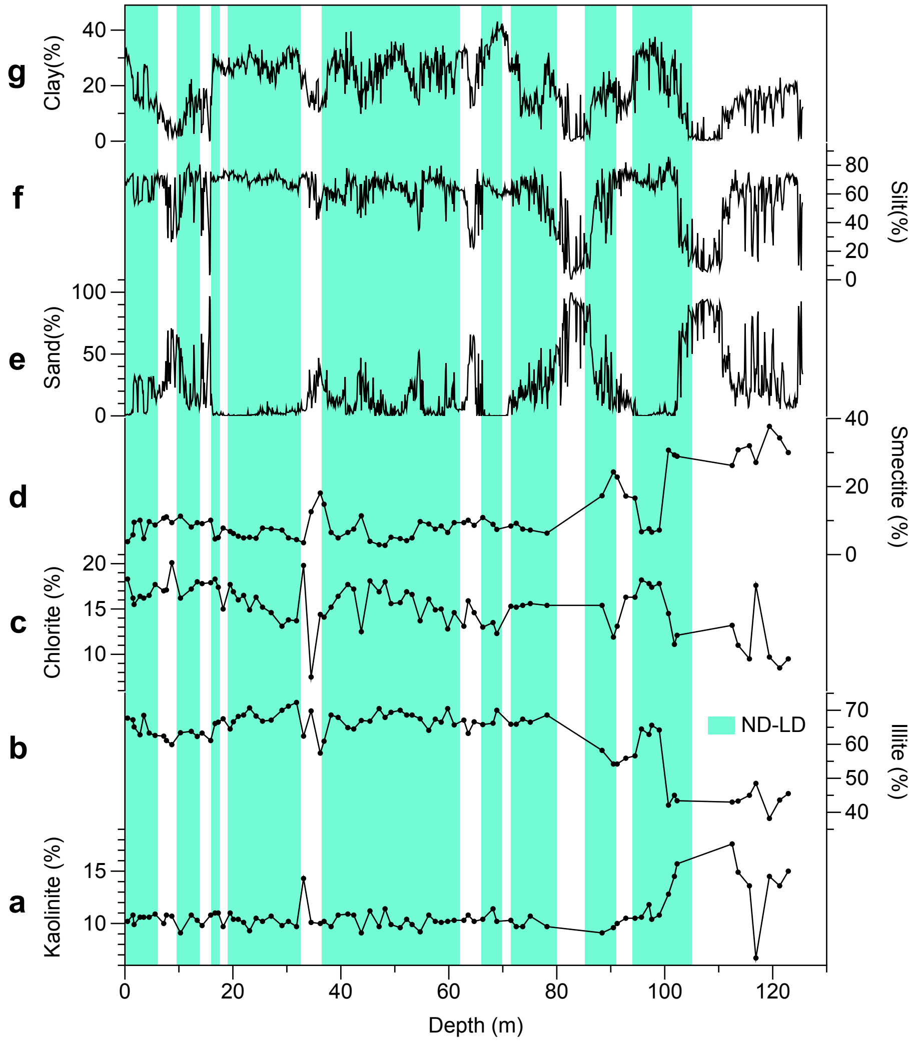

Supplementary Figure 2. Clay mineralogical and sedimentological parameters against depth in core NHH01. (a) Kaolinite. (b) Illite. (c) Chlorite. (d) Smectite. (e) Content of sand, (f) silt and (g) clay[16]. The green shades represent neritic-littoral deposits (ND-LD), while the blank area represents fluvial deposits in core NHH01.

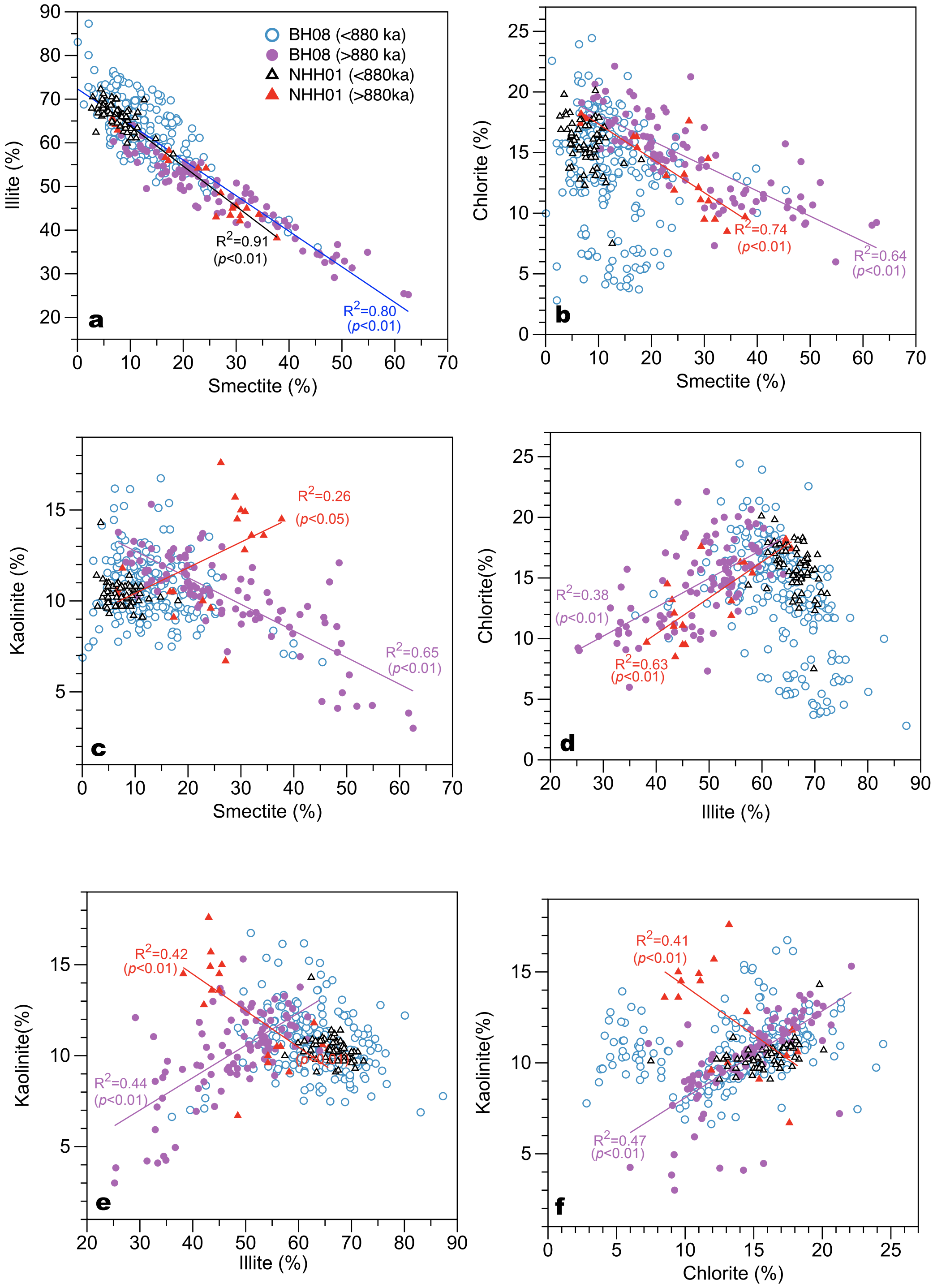

Supplementary Figure 3. Correlation of clay minerals for cores BH08 (circles) and NHH01 (triangles) separating at ~880 ka. (a) High correlation between illite and smectite for both cores. (b, c, d, e, f) Linear correlation, no matter whether it is positive or negative, for other clay mineral pairs exists only for samples prior to ~880 ka.

Supplementary References

1. Huanghe Water Conservancy Commission. *Bulletin of Huanghe Water Resources (In Chinese)*. Huanghe Water Conservancy Commission (2000).
2. China Ministry of Water Resources. *Chinese Rivers Sediment Bulletin (In Chinese)*. China Water & Power Press (2010).
3. Zhang, L. Y., Cai, Z., LI, Q. C. & Liu, P. Y. The runoff-silt relationship in Luanhe River Basin and effects of the water conservancy projects on the lower reaches. *Hubei Agric. Sci.* **51**, 3222-3225 (2012).
4. Zhang, J. Y. & Meng, X. J. Hydrological characteristics of the Liaoxi coast along the Bohai Sea (In Chinese). *Water conservancy and hydropower of the northeastern China* **22**, 13-14 (2004)
5. Compilation Committee of Records of Bays in China. *Records of Bays in China (In Chinese)*. China Ocean Press (1998).
6. China Ministry of Water Resources. *Chinese Rivers Sediment Bulletin (In Chinese)*. China Water & Power Press (2011).
7. Dou, Y. *et al.* Clay mineral distributions in surface sediments of the Liaodong Bay, Bohai Sea and surrounding river sediments: Sources and transport patterns. *Cont. Shelf Res.* **73**, 72-82 (2014).
8. Yang, S. Y., Jung, H. S., Lim, D. I. & Li, C. X. A review on the provenance discrimination of sediments in the Yellow Sea. *Earth Sci. Rev.* **63**, 93-120 (2003).

9. Li, J. *et al.* Provenance variations in the Holocene deposits from the southern Yellow Sea: Clay mineralogy evidence. *Cont. Shelf Res.* **90**, 41-51 (2014).
10. Li, Y., Li, A. C., Huang, P., Xu, F. J. & Zheng, X. F. Clay minerals in surface sediment of the north Yellow Sea and their implication to provenance and transportation. *Cont. Shelf Res.* **90**, 33-40 (2014).
11. Park, Y. A. & Khim, B. K. Origin and dispersal of recent clay minerals in the Yellow Sea. *Mar. Geol.* **104**, 205-213 (1992).
12. Shi, Y., Dai, X., Song, Z., Zhang, W. & Wang, L. Characteristics of clay mineral assemblages and their spatial distribution of Chinese Loess in different climatic zones. *Acta Sedimentolog. Sin.* **23**, 690-695 (2005).
13. Shi, X. F. *Chinese Marginal Seas: Marine Bottom Sediments*. China Ocean press (2012).
14. Yao, Z. Q. *et al.* Paleomagnetic and astronomical dating of sediment core BH08 from the Bohai Sea, China: Implications for glacial–interglacial sedimentation. *Palaeogeogr. Palaeoclimatol. Palaeoecol.* **393**, 90-101 (2014).
15. Shi, X. F. *et al.* Sedimentary architecture of the Bohai Sea China over the last 1 Ma and implications for sea-level changes. *Earth Planet. Sci. Lett.* **451**, 10-21 (2016).
16. Liu, J. *et al.* Magnetostratigraphy of a greigite-bearing core from the South Yellow Sea: Implications for remagnetization and sedimentation. *J. Geophys.l Res. Solid Earth* **119**, 7425-7441 (2014).
